# Pericentrin interacts with KASH domain-containing protein Syne-2

**DOI:** 10.1186/2046-2530-4-S1-P19

**Published:** 2015-07-13

**Authors:** N Falk, K Kessler, J Glöckner, K Boldt, M Ueffing, R Roepman, C Thiel, JH Brandstätter, A Gießl

**Affiliations:** 1 Department of Biology, Division of Animal Physiology, FAU Erlangen-Nürnberg, Erlangen, Germany; 2 Institute of Human Genetics, FAU Erlangen-Nürnberg, Erlangen, Germany; 3 Division of Experimental Ophthalmology and Medical Proteome Center, University of Tübingen, Tübingen, Germany; 4 Department of Human Genetics, Radboud University Nijmegen, Nijmegen, The Netherlands

## Objective

Pericentrin, a highly conserved protein of the pericentriolar material, serves as a multifunctional scaffold for numerous proteins and plays an important role in microtubule organization. Mutations in the human PCNT gene are associated with a range of diseases including primordial dwarfism and ciliopathies. In the mouse retina Pericentrin colocalizes with several proteins responsible for transport processes at the connecting cilium between the two photoreceptor compartments. In order to get more insights on the function of Pericentrin in the retina we try to identify new ciliary as well as centrosomal interaction partners.

## Methods

Identification of Pericentrin interaction partners was done by Tandem Affinity Purification, Yeast two-hybrid with a self-constructed cDNA library from mouse retina and GST pull-down.

## Results

We were able to show that Pericentrin interacts with Klarsicht/ANC-1/Syne-homologue (KASH) domain-containing protein Syne-2. Furthermore, we found a partial colocalization of Pericentrin and Syne-2 in the ciliary region of mouse photoreceptors. Pericentrin is localized at the basal body complex of the connecting cilium while Syne-2 seems to be localized in the whole inner segment of photoreceptors.

## Conclusion

Yu *et al*. (2010) suggested an interaction between Syne-2 and dynein/dynactin as well as kinesin complexes as the molecular motor of nuclear migration in the mouse retina. The interaction between Pericentrin and Syne-2 could play an essential role in interkinetic nuclear migration and may provide us new insights in the photoreceptor cell migration progress in general.

**Support: **DFG (GI770/1-1), Schmauser-Stiftung, Universitätsbund Erlangen-Nürnberg e.V.

